# Gypsum-calcium carbonate-gelatine composite graft for bone repair: Biocompatibility and histological assessment in a rat model

**DOI:** 10.1016/j.jobcr.2026.101559

**Published:** 2026-10-07

**Authors:** Anne Handrini Dewi, Andi Triawan, Mas Sahidayana Mohktar, Muhammad Akhsan Pridatama, Hersandy Dayu Kusuma, Fitri Nur Kayati, Aryan Morita, Retno Ardhani, Ananto Ali Alhasyimi

**Affiliations:** a Department of Dental Biomedical Sciences, Faculty of Dentistry, Universitas Gadjah Mada, Sleman, Yogyakarta, Indonesia; b Center of Excellence for Carbonate Apatite-based Extracellular Matrix and Adjuvant, Universitas Gadjah Mada, Sleman, Yogyakarta, Indonesia; c Department of Orthodontics, Faculty of Dentistry, Universitas Gadjah Mada, Sleman, Yogyakarta, Indonesia; d Department of Biomedical Engineering, Faculty of Engineering, Universiti Malaya, Federal Territory of Kuala Lumpur, Kuala Lumpur, Malaysia; e Department of Oral and Maxillofacial Surgery, Faculty of Dentistry, Universitas Gadjah Mada, Sleman, Yogyakarta, Indonesia; f Department of Chemistry, Faculty of Mathematics and Natural Sciences, Universitas Padjadjaran, Sumedang, Indonesia; g Department of Applied Science, Vocational School, Universitas Padjadjaran, Sumedang, Indonesia; h Research Center for Food Technology and Processing, National Research and Innovation Agency, Gunung Kidul, Yogyakarta, Indonesia

**Keywords:** Biocompatible implant, Regenerative bone therapy, Histological assessment

## Abstract

**Background:**

Gypsum (calcium sulphate dihydrate; POP) is a resorbable synthetic bone substitute with favourable biocompatibility; however, its rapid degradation limits clinical effectiveness. A modified composite incorporating calcium carbonate and gelatine hydrogel (POPHD) was developed to improve degradation control and osteogenic performance.

**Objective:**

This study aimed to evaluate and compare the biocompatibility and histological response of POP and POPHD in vitro and in vivo, with emphasis on cytotoxicity, soft-tissue response, and bone regeneration capacity.

**Methods:**

Cytotoxicity was assessed using the MTT assay on NIH3T3 fibroblast cells after 24 h of exposure. Twenty male Wistar rats were used for in vivo evaluation, comprising primary dermal irritation testing, subcutaneous implantation (14 days), and femoral condyle implantation (3 and 30 days). Histological sections were stained with haematoxylin-eosin and Mallory's trichrome and evaluated using validated grading scales.

**Results:**

Both materials demonstrated no cytotoxicity (cell viability >80%) and caused no skin irritation (PDII score 0). Subcutaneous implantation revealed comparable soft-tissue responses, though POPHD showed significantly greater angiogenesis (p = 0.003) and foreign body giant cell formation (p = 0.035), consistent with slower degradation. At 3 days post-femoral implantation, both groups showed similar early inflammatory responses. At 30 days, POPHD demonstrated superior bone formation quality and quantity (Score 3 vs Score 2) and enhanced angiogenic activity (p = 0.008) compared to POP.

**Conclusions:**

Modification of gypsum with calcium carbonate-gelatine hydrogel preserved biocompatibility while reducing degradation rate and promoting more advanced bone regeneration, suggesting POPHD as a promising synthetic bone graft substitute warranting further long-term investigation.

## Graphical abstract

Gelatine and CaCO_3_ were combined to form a hydrogel matrix, which was subsequently mixed with POP to produce cylindrical POP or POPHD specimens. In this study, the materials were subjected to in vitro cytotoxicity testing using NIH 3T3 fibroblast cells, in vivo subcutaneous implantation to assess tissue response, and femoral bone implantation to evaluate osteointegration and biocompatibility.

**Figure:**
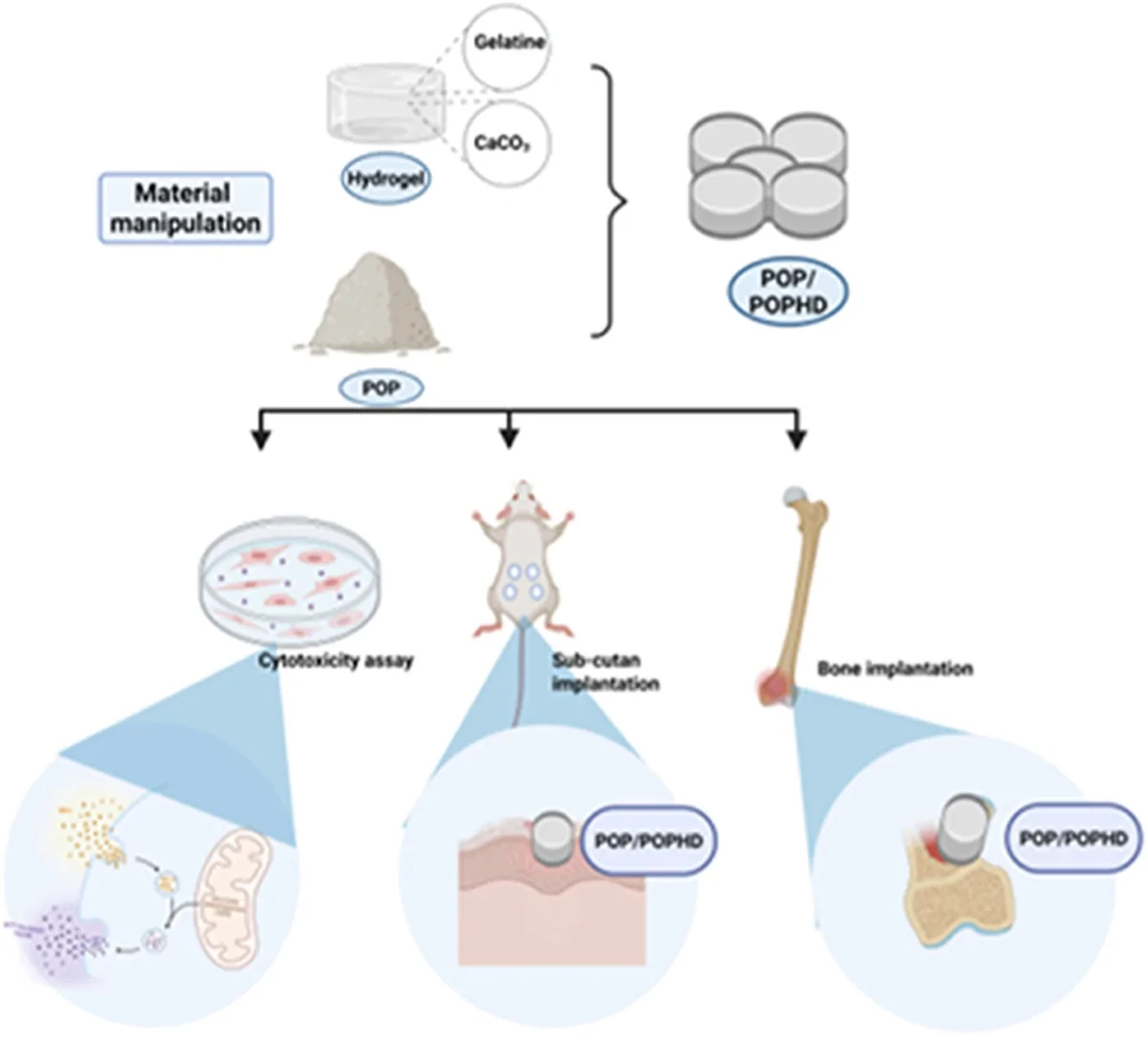

## 1 Introduction

Bone graft materials play a pivotal role in regenerative medicine by providing structural support and promoting bone repair in cases of fractures, defects, and reconstructive surgeries. In dentistry, bone grafting is commonly employed to manage alveolar bone deficiencies resulting from trauma, periodontal disease, tooth extraction, tumour resection, or for augmentation procedures such as sinus lift and pre-implant reconstruction.^1^ Traditionally, autografts, allografts, and xenografts have been utilised as gold standard for bone regeneration. However, these biological materials are associated with significant limitations, including donor site morbidity, immunogenic reactions, ethical constraints, and limited supply. Consequently, synthetic or alloplastic bone substitutes have gained increasing attention as viable alternatives. These materials exhibit favourable biocompatibility, consistent quality, and unlimited availability, while supporting osteoconduction and bone repair process.^2^^,^^3^

Among synthetic bone grafts, calcium-based ceramics remain the most widely applied due to their chemical similarity to the mineral phase of bone. Hydroxyapatite provides long-term structural stability; beta-tricalcium phosphate promotes rapid resorption and new bone formation; calcium sulphate offers both osteoconductivity and rapid degradation; while calcium carbonate exhibits moderate resorption and strong osteoconductive potential.^4^ The ability to tailor degradation kinetics and mechanical integrity makes calcium-based composites highly attractive for bone tissue engineering.^5^ Gypsum (calcium sulphate dihydrate) has resorbable, osteoconductive properties that provide temporary mechanical support and create space for new bone ingrowth.^6^ Calcium carbonate, a naturally occurring mineral, enhances bone mineralisation and facilitates cellular adhesion and proliferation.^7^ Hydrogels, with their high-water content and tuneable degradation, serve as biocompatible matrices capable of delivering bioactive agents and improving cell-material interaction.^8^ Thus, the combination of gypsum, calcium carbonate and a hydrogel offers a synergistic effect that mimics the native bone microenvironment and promotes osteointegration.

A critical aspect in the development of any bone substitute is biocompatibility. Following implantation, biomaterials elicit host immune responses that can modulate healing. The inflammatory phase is characterised by infiltration of neutrophils, macrophages, and lymphocytes, which secrete cytokines and growth factors regulating fibroblast activity, angiogenesis, and tissue remodelling.^9^ Although inflammation is essential for wound healing, excessive or chronic inflammatory responses may impair tissue integration.^10^ Therefore, understanding the cellular interactions and host response to implanted materials is essential for assessing their biocompatibility.^11^^,^^12^

The present study aims to evaluate the biocompatibility and histological response of a novel gypsum-calcium carbonate-hydrogel composite graft (POPHD) compared to gypsum alone (POP) using both in vitro and in vivo models. By integrating mineral and polymeric components, this composite is designed to enhance osteoconductivity, control degradation, and provide a favourable environment for bone regeneration.

## 2 Materials and methods

### 2.1 Gypsum-carbonate apatite hydrogel composite preparation

The composite was prepared according to a previous method^13^ with a modification to the crosslinking method. Calcium carbonate (CaCO_3_; Wako Pure Chemical Industries, Osaka, Japan) 2.5 g and 5.0 g type B gelatine from bovine bone (Nitta Gelatin Inc., Osaka, Japan) were mixed in distilled water. After 2 h of stirring, the pH was adjusted to 7.4, then freeze-dried for 3 days, and continued for dehydrothermal to induce crosslink formation at 140°C for 72 h. The bulk of calcium carbonate-gelatine composite was pulverised, sieved through a 150 μm mesh, and mixed with 50 wt% Plaster of Paris (POP) or gypsum (CaSO_4_.½H_2_O; Wako Pure Chemical Industries, Osaka, Japan) to prepare gypsum-calcium carbonate composite hydrogel (POPHD). The powders were subjected to a cell study and a primary dermal irritation test. For implantation, a cylindrical specimen was prepared by mixing water with powder at a ratio of 0.5, allowing it to set at 37°C for 24 h, and sterilising it with ethylene oxide gas (EOG).

### 2.2 Cytotoxicity evaluation on fibroblast culture cells

The NIH3T3 cell line was cultured in a complete medium containing Dulbecco's Modified Eagle Medium (DMEM; Invitrogen, Carlsbad, USA) supplemented with 10% bovine calf serum (Gibco, New York, USA), 1% penicillin–streptomycin (Gibco, New York, USA), and 0.5% amphotericin B (Gibco, New York, USA). The incubation environment was maintained at 37°C in a humidified 5% CO_2_ atmosphere. Upon reaching 80-90% confluence, the cells was detached using 0.25% trypsin-EDTA (Gibco) and the cells were then subcultured twice to obtain sufficient cell number. For cytotoxicity evaluation, we seeded the cells at a density of 1 × 10^4^ cells/well in a 96-well plate. After 24 h of incubation to allow monolayer formation, the culture medium was replaced with 100 μL fresh culture medium containing 0.05%, 0.1%, or 0.2% POP or POPHD, and the cells for the next 24 h. To evaluate the viability of the cells, we applied 1 mg/ml of 3-[4,5-dimethylthiazol-2-yl]-2,5-diphenyl tetrazolium bromide (MTT) reagent at a 1:10 v/v ratio into each well, then we incubated the cells for 2 h. During the incubation, living cells converted the MTT reagent into an insoluble formazan crystal. We replaced the culture medium with 100 μL DMSO to dissolve the formazan crystals, resulting in a colour change in the solution. The optical density (OD) was then measured with a microplate reader (Multiskan Sky, Thermo Scientific) at 570 nm. The OD results were compared across the sample groups relative to the only cell group, expressed in percentage and an 80% threshold to determine cytotoxicity. For statistical analysis, the experimental data were collected from 5 replications.

### 2.3 In vivo study on the Wistar rat model

The Ethics Committee of the Faculty of Dentistry Universitas Gadjah Mada approved the protocol before animal study initiation (No. 0103/KE/FKG-UGM/EC/2022). Twenty healthy adult male Wistar rats (3-4 months old, 250-350 g) were acclimatised for 7 days before the experiment with free access to food and water under a 12-h light/dark cycle. We applied the tested materials on the skin surface, subcutaneous tissue, and bone under total anaesthesia. The procedure involved intramuscularly administering a cocktail of ketamine (80-100 mg/kg) (OGBDexa, Banten, Indonesia) and xylazine (5-10 mg/kg) (Xyla, Interchemie, Netherland).

### 2.4 Topical material application on the dorsal skin

The potential of the tested materials was evaluated to induce an acute reaction using the primary dermal irritation test on the dorsal skin of the rats. A total of 5 rats were used, and each rat received 4 compared substances. Unlike in the previous method,^14^ in which 0.5 g solid or 0.5 ml liquid substances were applied, the amount to the limited area was adjusted on the dorsal rat skin. Briefly, 0.05 g of POP and POPHD were applied directly to the shaved and cleaned skin surface. As negative and positive controls, 0.05 ml of saline and 1% sodium lauryl sulphate (SLS; Himedia, Thane, India) solution were administered, respectively. The application site was covered with a gauze, and after 4 h, the skin was exposed and cleaned. We observed erythema (redness) and oedema (swelling) at 1, 24, 48, and 72 h following material removal. They were scored according to the numerical Draize technique for dermal reaction (Table 1).^15^

**Table 1:** The Draize score for dermal reaction upon direct contact with a substance.

Clinical Findings	Scores
**Erythema and eschar tissue formation**	
No erythema	0
Very slight erythema (barely perceptible)	1
Well-defined erythema	2
Moderate erythema	3
Severe erythema to eschar tissue formation on the lesion, preventing observation of erythema	4

**Oedema formation**	
No oedema	0
Very slight oedema (barely perceptible)	1
Slight oedema (well-defined edges by definite tissue raising)	2
Moderate oedema (tissue raised approximately 1 mm)	3
Severe oedema (tissue raised more than 1 mm and extending beyond the area of exposure	4

In the last step, the primary dermal irritation index (PDII) was calculate by applying the Draize score to the equation:

$$\text{Primary}\;\text{dermal}\;\text{irritation}\;\text{index}=\frac{Sum\;of\;mean\;of\;redness\;and\;oedema\;from\;1,24,48,and\;72\;hours\;readings}{Number\;of\;test\;sites\;x\;scoring\;intervals\;x\;number\;of\;replication}$$

The PDII indicates the level of skin reaction to materials, starting from non-irritating (score 0), slightly irritating (score >0 – 2.0), moderately irritating (score 2.1 – 5.0), and severely irritating (score >5.0).

### 2.5 Material implantation into the subcutaneous pocket

The soft-tissue response to POP and POPHD was studied after 14 days of subcutaneous implantation on the dorsal region of five rats. In this procedure, the skin was shaved and disinfected with povidone-iodine (OneMed, Sidoarjo, Indonesia) after administering the general anaesthesia. A 1 cm longitudinal incision was performed parallel to the vertebral column on both the left and right sides, creating subcutaneous pockets. One cylindrical specimen (POP or POPHD) was inserted into each pocket. The pocket was closed using 4/0 catgut suture (OneMed, Jiangsu, China) on the intradermal layer and non-resorbable 4/0 silk braided suture (OneMed, Jiangsu, China) for skin closure. After 14 days, the rats were euthanised using the ketamine-xylazine overdose method for skin tissue harvesting. The implant and surrounding tissue were dissected and fixed together in 10% buffered formalin pH 7 (Surgipath Leica, Richmond, USA). For histological evaluation, tissue sections of 3 μm thickness were prepared and stained with haematoxylin and eosin (HE). Three regions were evaluated: the soft tissue surrounding the materials, the material-soft tissue interface, and the material region, under a stereo microscope (Olympus SZ2-ILST, Olympus Corp., Tokyo, Japan) and a light microscope (Nikon YS100, Nikon Corp., Tokyo, Japan) equipped with an Optilab imaging system (Miconos, Yogyakarta, Indonesia). Observations were initiated at low (4 × 10 × 1.6), then medium (10 × 10 × 1.6), and finally high (40 × 10 × 1.6) magnification. A histological grading scale for soft-tissue reaction to material implantation (Table 2) and the percentage of material degradation were determined based on these observations. Each degradation criterion was scored on a scale of 0 to 4: score 0 if the material was intact, score 1 for >0%–25% degradation, score 2 for >25%–50% degradation, score 3 for >50%–75% degradation, and score 4 for >75%–100% degradation. For comparison, the median score of five replications was calculated. The number of newly formed blood vessels and multinucleated foreign body giant cells (FBGCs) was counted from five fields of view within the regions of interest at the interstitial area under 40 × 10 × 1.6 magnification. The average values across five rats were then compared.

**Table 2:** Histological grading scale for soft-tissue reaction to a material implantation.

Soft-tissue reaction	Scores
**Capsule thickness rating**	
Not observed	0
>30 fibroblasts	1
10-30 fibroblasts	2
5-9 fibroblasts	3
1-4 fibroblasts	4

**Capsule quality**	
Cannot be evaluated because of infection or factors not necessarily related to the material	0
Consists of masses of inflammatory cells with little or no signs of connective tissue organisation	1
Granulous and dense, containing both fibroblasts and many inflammatory cells	2
Fibrous, but immature, showing fibroblasts and little collagen	3
Fibrous, mature, not dense, resembling connective or fat tissue in the non injured regions	4

**Material-tissue interface quality: foreign body reaction**	
Cannot be evaluated because of infection or factors not necessarily related to the material	0
Multiple layers of macrophages with presented foreign body giant cells (FBGCs)	1
One layer of macrophages with presented FBGCs	2
Scattered foci of macrophages and presented FBGCs	3
Fibroblasts contact the implant surface without the presence of macrophages or FBGCs	4

**Material-tissue interface quality: cell infiltration**	
Cannot be evaluated because of infection or factors not necessarily related to the material	0
Tissue is dense and exclusively of an inflammatory type	1
Shows giant cells and other inflammatory cells in abundance, but a connective tissue component in between	2
Shows blood vessels and young fibroblasts invading the space. Few macrophages may be present.	3
Tissue is fibrous, mature, not dense, resembling the connective or fat tissue in non-injured regions	4

### 2.6 Material implantation into the femoral condyle

Bone tissue response to materials was observed at 3 and 30 days after implantation in five rats that received both POP and POPHD. The lateral surface of both right and left femoral condyles was shaved and disinfected using povidone-iodine, followed by a 1-cm longitudinal incision to expose the bone surface. A standardised bone defect measuring 2 mm in diameter by 3 mm in depth was created using an implant bur (Osstem, Busan, South Korea) operated at 1000 rpm with 65 N cm torque under continuous saline irrigation to prevent thermal injury. A cylindrical specimen of POP or POPHD was inserted into the femoral bone defect, and the muscle and skin layers were closed with resorbable and non-resorbable sutures, respectively. Postoperatively, enrofloxacin 100 mg/ml (Interflox-100, Interchemie, Maastricht, the Netherlands) was administered intramuscularly at a dose of 10 mg/kg body weight daily to prevent infection. The rats were observed daily to assess overall welfare and healing progress, including activity level, eating behaviour, wound closure, and signs of inflammation. Following euthanasia, femoral bone tissue was harvested. The collected tissues were fixed in 10% buffered formalin (pH 7.0) for 24 h, then subjected to decalcification in 10% EDTA (Titriplex III, Merck, Darmstadt, Germany) for one month, followed by paraffin embedding, sectioning at 3 μm thickness, and histochemical staining with haematoxylin-eosin (HE) and Mallory's trichrome. Neutrophil and lymphocyte infiltration, fibrous capsule formation, vascularisation, and FBGCs were evaluated under a light microscope (Nikon YS100, Nikon Corp., Tokyo, Japan) equipped with an imaging system (Optilab, Miconos, Yogyakarta, Indonesia). Hard tissue reaction was scored according to a histological grading scale comprising qualitative and quantitative evaluations of bone formation and the material-tissue interface (Table 3). Material degradation was evaluated using a stereo microscope (Olympus SZ2-ILST, Olympus Corp., Tokyo, Japan) with the same scoring rubric applied in the subcutaneous implantation experiment.

**Table 3:** Histology grading scale for hard-tissue reaction to a material implantation.

Hard-tissue reaction	Scores
**Defect closure quantity: bone formation**	
No bone fill/fibrous tissue	0
0% - <25% bone closure	1
25% - <50% bone closure	2
50% - <75% bone closure	3
75% - 100% bone closure	4

**Defect closure quality: bone formation**	
Inflammatory cell infiltration	0
Other tissue than bone (e.g. fibrous tissue)	1
Lamellar or woven bone with bone-forming activity and osteoclastic activity	2
Lamellar or woven bone with bone-forming activity	3
Similar to the native cortical bone	4

**Material-tissue interface quality**	
Severe inflammation	0
Fibrous tissue capsule formation, heavy inflammation	1
Localised fibrous tissue not arranged as a capsule, moderate inflammation	2
Remodelling lacuna with osteoblasts and/or osteoclasts at the surface, minimal inflammation	3
Direct bone-to-implant contact without a soft tissue interlayer, no inflammation	4

**Material-tissue interface quality**	
The implant cannot be evaluated because of problems that may not only be related to the material to be tested, severe inflammation, degradation-related damage (e.g. osteolysis)	0
Tissue in the interface consists of fibrous connective tissue with a pronounced cellular and vascular component, and heavy inflammation	1
Tissue in the interface consists of fibrous connective tissue characterised by collagen fibres condensation, immature bone, and moderate inflammation	2
Bone formation can be observed in the interface with minimal inflammation	3
Mature bone and differentiation of bone marrow can be observed without implant remnants	4

#### 2.6.1 Data analysis

The results of the experimental groups were compared using a statistical software (IBM SPSS Statistics 25, IBM Inc., New York, US). The parametric *t*-test and the nonparametric Kruskal-Wallis tests were applied at a 95% level of significance.

## 3 Results

### 3.1 Cytotoxicity and primary dermal irritation

Fibroblast NIH3T3 cells withstood 0.05%, 0.1%, and 0.2% POP or POPHD in the culture medium for 24 h with no viability percentage below 80%, as illustrated in Fig. 1, indicating negative cytotoxicity of both materials. The bar charts and boxplots showed a relatively uniform distribution of OD values, with several outliers in the 0.1% and 0.05% POP, as well as in the 0.1 and 0.2 POPHD groups. Further analysis showed homogenous distributions of data and reproducibility across replicates, with minor outliers likely attributable to biological variability or minor technical deviations during culture. The one-way ANOVA yielded an F value of 0.478 (p = 0.789), indicating no significant differences among groups. This finding indicates that both POP and POPHD are biocompatible, thus safe within the evaluated concentration range.

**Fig. 1:**
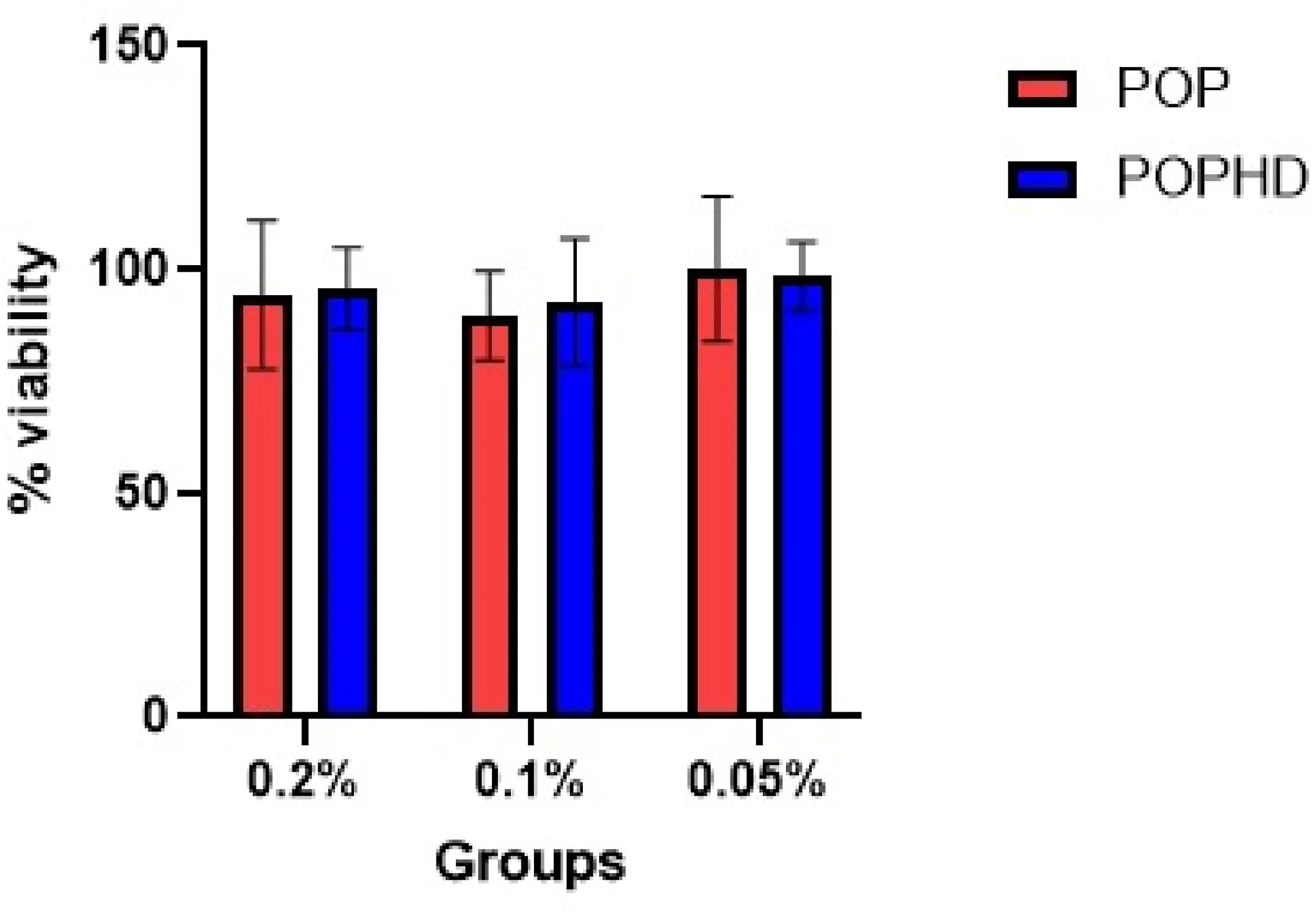
Percentages of viability of the POP and POPHD groups at the concentrations of 0.2%, 0.1%, and 0.05% after 24 h of fibroblast NIH3T3 culture are more than 80%.

Neither POP nor POPHD induced skin irritation. No visible signs of erythema or oedema were observed on the skin at 1, 24, 48, or 72 h after 4 h of contact with POP and POPHD (PDII score 0). This finding was similar to the area that was given saline (PDII score 0). However, as suggested in the previous study,^16^ SLS was slightly irritating (PDII score 0.7).

### 3.2 Soft-tissue response and material degradation following subcutaneous implantation

Histological evaluation after 14 days of subcutaneous implantation revealed similar soft-tissue reactions in both POP and POPHD groups (Fig. 2). The surrounding soft-tissue matrix consisted of fibrous, mature, non-dense connective tissue resembling native dermis with well-organised fibroblasts (Score 4), moderate fibroblast density (5–9 fibroblasts; Score 3), scattered macrophages with foreign body cells (Score 3), and neovascularisation with fibroblast infiltration in the interstitial regions (Score 3). Quantitative analysis revealed differences between the two groups. The POPHD group exhibited significantly greater angiogenesis (21.00 ± 1.87) compared to POP (17.00 ± 1.00; p = 0.003). The number of multinucleated FBGCs was also significantly higher in the POPHD group (18.80 ± 1.64) than in POP (15.60 ± 2.30; p = 0.035). Material degradation scoring indicated slower degradation in POPHD (Score 2) compared to POP (Score 3).

**Fig. 2:**
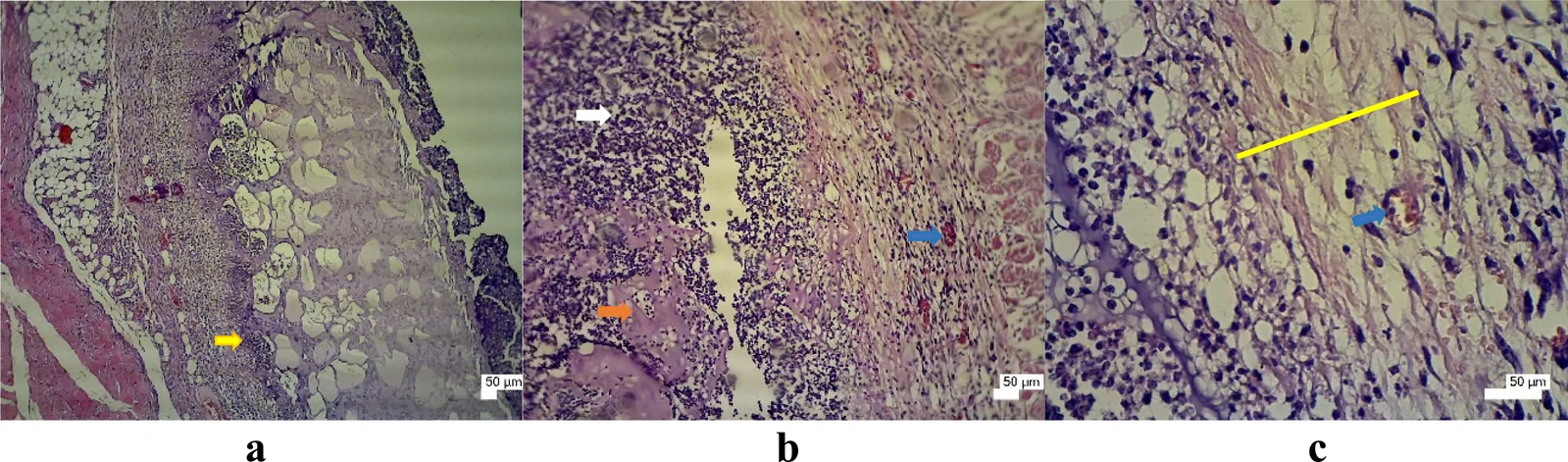
**Representative histological images of subcutaneous tissue surrounding the implanted scaffold after 14 days.** (a) Low-magnification view (4×13) showing the fibrous capsule (yellow arrow) demarcating the implant material from the surrounding tissue. (b) Intermediate magnification (10×13) highlighting numerous inflammatory cells (white arrows), newly formed blood vessels (blue arrows), and remnants of implant material (orange arrow). (c) High-magnification image (40×13) showing the fibrous capsule (yellow line) with newly developed blood vessels (blue arrow) interspersed among collagen fibers. All sections were stained with haematoxylin and eosin (HE). Scale bar = 50 μm. (For interpretation of the references to colour in this figure legend, the reader is referred to the Web version of this article.)

### 3.3 Bone tissue response following femoral condyle implantation

In the femoral condyle implantation model, both the POP and the POPHD groups (Fig. 3) exhibited limited new bone formation at 3 days post-surgery (Score 1). Histological examination revealed that fibrous connective tissue occupied the peripheral regions of the bone defects in both groups (Score 1), with no evident mineralized bone matrix formation. The defects were still dominated by inflammatory cell infiltration (Score 1), indicating active early tissue response. At the bone-implant interface, both groups displayed fibrous capsule formation, accompanied by marked inflammatory cell presence (Score 1), characteristic of acute response to degradable biomaterials. The interstitial regions in both groups (Score 1) contained fibrous connective tissue with abundant inflammatory and vascular components, reflecting a pronounced but controlled inflammatory reaction. This is consistent with the early inflammatory phase of bone healing.^10^

**Fig. 3:**
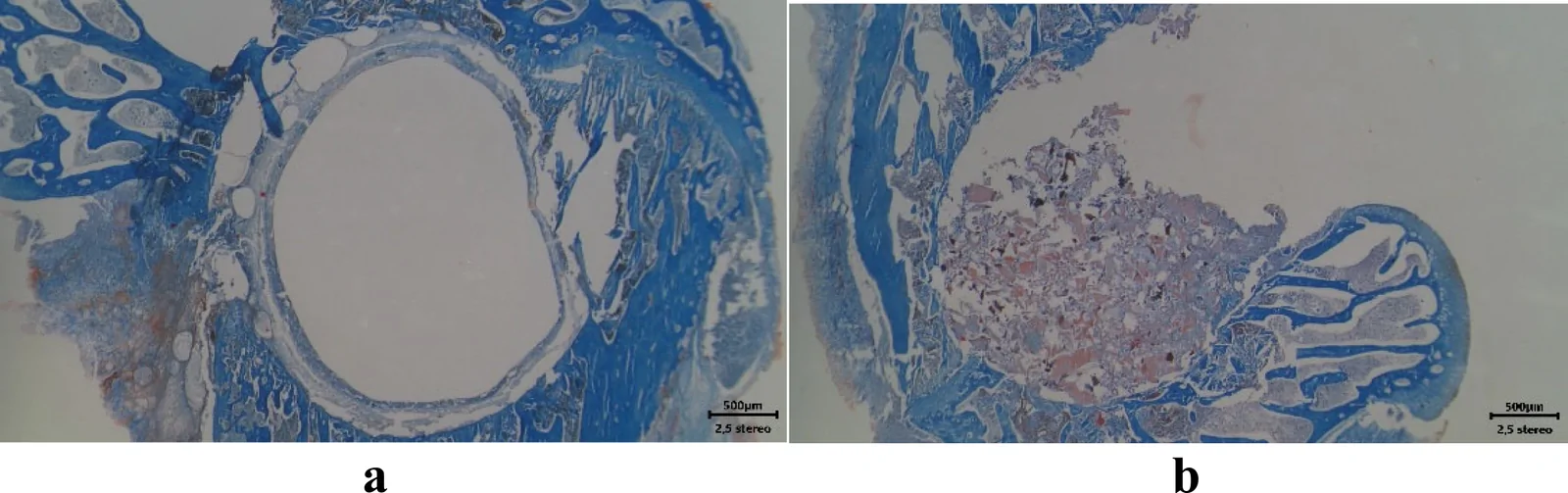
**Histological appearance of femoral condyle defects after 3 days of implantation**. (a) defect treated with Plaster of Paris (POP), and (b) defect treated with the gypsum–calcium carbonate–hydrogel composite (POPHD). Specimens were paraffin-embedded, sectioned, and stained with Mallory's trichrome. Images were obtained using a stereomicroscope at 2.5×10 magnification. Scale bar = 500 μm.

This study found that neither POP nor POPHD halted the inflammatory process. The number of neutrophils, as the first responder at the site of acute inflammation, recruited into the defect region in the POP and POPHD was (50.67 ± 5.46) and (45.33 ± 6.35), respectively, without a statistically significant difference (p = 0.202) at 3 days post-implantation. Similarly, the POP group showed a higher number of blood vessels (9.60 ± 1.82) than the POPHD group (9.40 ± 1.14), but this difference was not statistically significant (p = 0.840). Successful bone regeneration is associated with controlled inflammation from the earliest stage. It was proposed that an immunomodulation strategy has the potential to achieve bone healing in both acute and chronic inflammation.^17^ Both POP or POPHD in this study induced a favourable inflammatory response that did not interfere with bone healing (Fig. 4). At 30 days post-implantation, the POPHD group had a significantly (p = 0.008) higher number of lymphocyte infiltrates (177.60 ± 1.82) than the POP group (127.60 ± 3.58). At the same time, the number of FBGCs was slightly higher, but statistically insignificant (p = 0.151), in the POPHD group (1.60 ± 1.14) than in the POP (0.60 ± 0.55), reflecting a mild, localised foreign-body reaction associated with the natural degradation of the implant. This phenomenon was observed simultaneously with a lower implant degradation percentage in the POPHD group (Score 2) than in the POP group (Score 3).

**Fig. 4:**
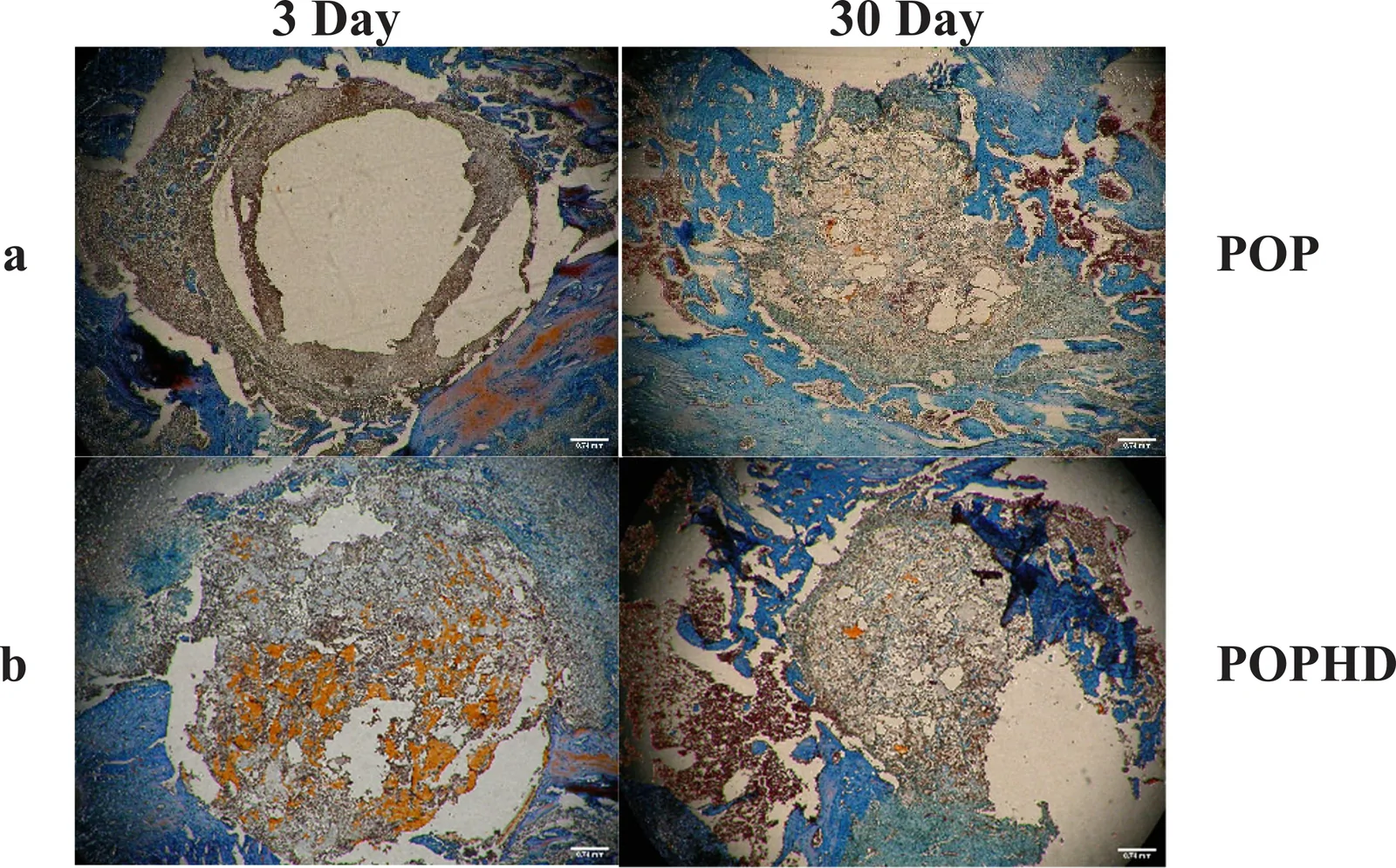
**Histological evaluation of bone regeneration in femoral condyle defects at 3 and 30 days post-implantation.** (a) defect treated with Plaster of Paris (POP), and (b) defect treated with the gypsum–calcium carbonate–hydrogel composite (POPHD). Sections were paraffin-embedded and stained with Mallory's trichrome to distinguish mineralized bone (red) and connective tissue (blue). Images were captured under a stereomicroscope at 4×13 magnification. Scale bar = 500 μm. (For interpretation of the references to colour in this figure legend, the reader is referred to the Web version of this article.)

All histological grading parameters for bone reactions to material implantation were increased in both groups at 30 days post-implantation. However, the POPHD group showed superior scores for bone formation both in quality and quantity (Score 3), compared with the POP group (Score 2). A similar trend was also observed for the material-tissue interface quality, with the POPHD group showing a more structured osteogenic matrix (Score 3), compared to fibrous tissue with immature bone in the POP group (Score 2). Remodelling lacunae with osteoblasts and/or osteoclasts were identified at the surface on both groups (Score 3).

Our findings showed that angiogenesis supported more progressive bone formation in the POPHD group. The POPHD group showed significantly (p = 0.008) enhanced angiogenic activity, as evidenced by a higher number of blood vessels (33.00 ± 1.87) compared to the POP (25.40 ± 2.97). New blood vessels support the provision of oxygen, nutrients, and pro-osteogenic factors, which are essential in bone regeneration. Impeding angiogenesis was shown to lead to the failure of bone formation. Inversely, strategies to improve angiogenesis during the repair phase were noted as promising for bone defect therapy.^18^

## 4 Discussion

A biodegradable composite has been widely explored as a substitute for autologous bone graft, and some products are available in the market. However, this is not the end of the quest to find the best replacement for an autologous bone graft, because none of the available alternatives can currently compete with it.^19^ In developing a novel autologous bone graft substitute, material selection, preparation, and design must be followed by biological tests to establish a safety database. Moreover, the tests are important for demonstrating the material's potential clinical significance. In this study, we observed that POP and POPHD appeared to be noncytotoxic after 24 h of interaction with cells. Undetected skin irritation after 4 h of a direct contact also supported this biocompatibility evidence. In a positive case, a substance triggers inflammation upon contact as indicated by erythema and/or oedema on the skin. Unlike an allergy, irritation can be clinically manifested without prior contact and is more closely related to the inherent harm of the material.^20^ In this study, the modification of POP by incorporating gelatine-CaCO3 did not alter its negative potential to induce skin irritation upon direct contact, which was equal to that of saline.

The present study evaluated the biocompatibility and bone regenerative potential of gypsum (POP) and its modified composite (POPHD) through a stepwise in vitro and in vivo approach. POP and POPHD demonstrated no cytotoxicity against NIH3T3 fibroblast cells and caused no primary dermal irritation, confirming their biocompatibility. In a positive case, a substance triggers inflammation upon contact as indicated by erythema and/or oedema on the skin. Unlike an allergy, irritation can be clinically manifested without prior contact and is more closely related to the inherent harm of the material.^20^ The incorporation of gelatine-CaCO_3_ into POP did not alter this favourable safety profile, as POPHD produced a PDII score equivalent to that of saline. Both POP and POPHD contain calcium ion, which stimulates angiogenesis via vascular endothelial growth factor (VEGF) regulation.^21^ An in vitro study confirmed that calcium modification of calcium ions increases angiogenic capacity and the VEGF release.^22^ However, POPHD was found to have advantages due to its gelatine content. A previous study showed that the degradation products of gelatine increased the number of new blood vessels in vivo, and this increase was proportional to the increase in gelatine concentration^23^**.** Calcium not only contributes to angiogenesis via VEGF regulation during bone regeneration, but also in osteoimmunomodulation. As an immunomodulator, calcium released from a biodegradable graft activates a signalling pathway that polarises macrophages towards a pro-regeneration subset (M2)^24^**.** The M2 macrophages directly regulate bone regeneration by influencing the behaviour of mesenchymal stem cells, osteoblasts, and osteoclasts towards a homeostatic process.^25^ A study showed that an exosome from an engineered M2 macrophage upregulated VEGF secretion of bone marrow stem cells in vitro. In vivo, the M2 macrophage intensifies the recruitment of the native bone marrow stem cells as well as inducing their osteogenic differentiation.^26^

Subcutaneous and femoral bone implantation in this study showed that POP and POPHD elicited a similar grade of inflammation. However, macrophage fusion into FBGCs was more evident in the POPHD group, accompanied by delayed biodegradability. The addition of calcium carbonate was shown to decrease the solubility and disintegration of calcium sulphate-based materials, thus delaying degradation^27^**.** This approach simply involves combining a low-dissolution material into POP. Thus, total degradation can be controlled by manipulating the proportion of the additive, including calcium carbonate.^28^ Despite the promising findings, several limitations should be acknowledged. First, the observation period was limited to 30 days, which may not fully capture the complete bone regeneration cycle or long-term scaffold-tissue interactions. Second, the sample size was small (n = 5 per group), which may limit statistical power and generalisability. Third, no empty defect control group was included, making it difficult to establish baseline spontaneous healing capacity. Fourth, functional assessments such as micro-computed tomography and biomechanical testing were not performed, precluding quantitative evaluation of bone volume and mechanical integrity. Future studies incorporating longer observation periods, larger sample sizes, and multimodal evaluation are recommended to validate the clinical translational potential of POPHD.^29^

## 5 Conclusion

The modification of gypsum with a calcium carbonate-gelatine hydrogel reduces its in vivo degradability without provoking skin irritation and uncontrolled inflammation. The composite also offers an advantage by promoting a significant new blood vessel formation and more advanced bone formation than that after gypsum implantation.

## Ethical clearance

This study was approved by the Health Research Ethics Committee, Faculty of Dentistry, Universitas Gadjah Mada, Indonesia (Approval No. 0103/KE/FKG-UGM/EC/2022.). All procedures followed institutional guidelines for the care and use of laboratory animals and complied with the National Guidelines for the Care and Use of Laboratory Animals in Indonesia and the internationally accepted principles for laboratory animal use and care.

## Funding

The authors gratefully acknowledge the funding support from “Improvement of Doctoral Competency Program 2025” Research Grant, the 10.13039/501100012521Universitas Gadjah Mada (Contract No. 1618/UN1/DITLIT/PT.01.00/2025) and “Dana Masyarakat” Grant from the Faculty of Dentistry, Universitas Gadjah Mada (Contract No. 3789/UN1/KG/PT/2025).

## Declaration of competing interest

The authors declare that they have no known competing financial interests or personal relationships that could have appeared to influence the work reported in this paper.
